# Antimicrobial Activity of Human Fetal Membranes: From Biological Function to Clinical Use

**DOI:** 10.3389/fbioe.2021.691522

**Published:** 2021-05-31

**Authors:** Taja Železnik Ramuta, Tina Šket, Marjanca Starčič Erjavec, Mateja Erdani Kreft

**Affiliations:** ^1^Institute of Cell Biology, Faculty of Medicine, University of Ljubljana, Ljubljana, Slovenia; ^2^Department of Synthetic Biology and Immunology, National Institute of Chemistry, Ljubljana, Slovenia; ^3^Department of Biology, Biotechnical Faculty, University of Ljubljana, Ljubljana, Slovenia

**Keywords:** fetal membrane, perinatal derivatives, amniotic membrane, amnio-chorionic membrane, placenta, antimicrobial peptides, bacteria, antibacterial activity

## Abstract

The fetal membranes provide a supportive environment for the growing embryo and later fetus. Due to their versatile properties, the use of fetal membranes in tissue engineering and regenerative medicine is increasing in recent years. Moreover, as microbial infections present a crucial complication in various treatments, their antimicrobial properties are gaining more attention. The antimicrobial peptides (AMPs) are secreted by cells from various perinatal derivatives, including human amnio-chorionic membrane (hACM), human amniotic membrane (hAM), and human chorionic membrane (hCM). By exhibiting antibacterial, antifungal, antiviral, and antiprotozoal activities and immunomodulatory activities, they contribute to ensuring a healthy pregnancy and preventing complications. Several research groups investigated the antimicrobial properties of hACM, hAM, and hCM and their derivatives. These studies advanced basic knowledge of antimicrobial properties of perinatal derivatives and also provided an important insight into the potential of utilizing their antimicrobial properties in a clinical setting. After surveying the studies presenting assays on antimicrobial activity of hACM, hAM, and hCM, we identified several considerations to be taken into account when planning future studies and eventual translation of fetal membranes and their derivatives as antimicrobial agents from bench to bedside. Namely, (1) the standardization of hACM, hAM, and hCM preparation to guarantee rigorous antimicrobial activity, (2) standardization of the antimicrobial susceptibility testing methods to enable comparison of results between various studies, (3) investigation of the antimicrobial properties of fetal membranes and their derivatives in the *in vivo* setting, and (4) designation of donor criteria that enable the optimal donor selection. By taking these considerations into account, future studies will provide crucial information that will enable reaching the optimal treatment outcomes using the fetal membranes and their derivatives as antimicrobial agents.

## Introduction

Fetal membranes, namely human amnio-chorionic membrane (hACM), human amniotic membrane (hAM) and human chorionic membrane (hCM) have been used in clinic for several decades. hAM has been used most commonly in ophthalmology for ocular surface reconstruction (Riau et al., 2010; Cirman et al., 2014; Malhotra and Jain, 2014; Jirsova and Jones, 2017) and in dermatology for treatment of burns, chronic wounds and ulcers (Castellanos et al., 2017; Farhadihosseinabadi et al., 2018). hACM and hCM have also been used to improve wound healing, although less commonly than hAM (Zelen et al., 2015; Tenenhaus, 2017; Kogan et al., 2018). In the last years, progress has been made in the field of basic knowledge regarding the antimicrobial activity of fetal membranes (King et al., 2007a,b; Klaffenbach et al., 2011). Since the use of fetal membranes in tissue engineering and regenerative medicine is increasing (Lim, 2017; Nejad et al., 2021) and microbial infections represent a crucial complication in various treatments (Ikada, 2006; Kuijer et al., 2007; Guo and Dipietro, 2010; Deptuła et al., 2019), antimicrobial properties of fetal membranes are attracting more and more attention. In this review, we focus on the biological function of hACM, hAM, and hCM, their antimicrobial properties, and the potential of their use in clinical applications.

## The Biological Function of Fetal Membranes

The fetal membranes are composed of hAM and hCM. Their role is to surround the embryo and later fetus during gestation and they are crucial for maintaining a pregnancy to delivery (Bryant-Greenwood, 1998; Verbruggen et al., 2017). Moreover, the amniotic sac is surrounded by outer hCM and hAM on the inside, and together they provide a supportive environment for the growing fetus. hAM is in direct contact with the human amniotic fluid (hAF), in which the embryo or fetus is developing, allowing it to sense and respond to the needs of the fetus. Furthermore, fetal membranes shield the fetus from environmental and endogenous hazards, such as physical, chemical, or biological changes that may harm the fetus (Mamede et al., 2012).

Firstly, the fetal membranes must bear the hydrostatic pressure of the hAF, whose volume changes with gestation from approximately 10 ml at week 8 up to approximately 1000 ml at 34 weeks, and dropping slightly until birth (Brace and Wolf, 1989). In the third trimester, the hAF volume is maintained mainly by regulating the rate of absorption through the amnion into fetal blood. In details, it is controlled based on the ratio of fetal urine component that acts as a stimulator for absorption and a fetal membrane-derived inhibitor (Anderson et al., 2013; Brace et al., 2013, 2018). Secondly, the membranes must also withstand sudden impacts and compresses, for instance from fetal movements and Braxton-Hicks contractions. Although hAM is approximately five times thinner, it is up to ten times stronger and stiffer than hCM (Oxlund et al., 1990). Preterm membranes have been shown to be stronger than term membranes, which is the result of the regulated physiological process of membrane weakening, since the membrane should rupture during labor (Moore et al., 2006; Joyce et al., 2009; Rangaswamy et al., 2011). Thirdly, there is evidence that hAM regulates the pH of the hAF by converting bicarbonate to the CO_2_ with human carbonic anhydrase isoenzymes, which are strongly expressed in human amniotic epithelial cells (hAEC) (Mühlhauser et al., 1994).

Importantly, the hACM normally protects the fetus from pathogens that can directly or indirectly induce dangerous pregnancy complications, namely premature rupture of membranes and preterm delivery (Gonçalves et al., 2002; Romero et al., 2003, 2006). Membranes provide such protection in several ways, the first being structural impermeability to pathogens. Cells in the membranes also express antimicrobial peptides (AMPs) that directly target microorganisms in the hACM or hAF (Zhang et al., 2001; King et al., 2007b; Stock et al., 2007).

**FIGURE 1:**
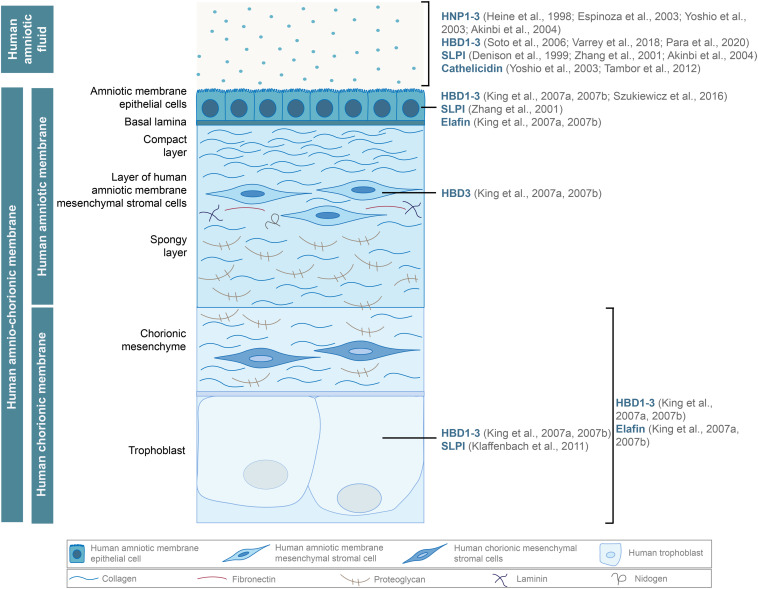
Antimicrobial peptides, such as human α (HNP1-3) and β-defensins (HBD1-3), cathelicidin, and proteases of the WFDC family (SLPI, elafin), are secreted by the hAM and hCM cells and found in the hAF. Adapted from Cirman et al. (2018).

Nevertheless, if intra-amniotic infections occur, the fetal membranes and hAF are largely involved in activating and regulating an immune response (Figure 1). This is evidenced by an increased presence of immune cells, upregulated AMP’s, cytokines and chemokines, activated pattern recognition receptors that trigger inflammatory signaling pathways, and changes in the extracellular matrix of the hAM (Gillaux et al., 2011; Romero et al., 2016; Myntti et al., 2017; Gomez-Lopez et al., 2019; Bhatti et al., 2020; Galaz et al., 2020; Gomez-Lopez et al., 2020).

## Antimicrobial Peptides in Fetal Membranes

Antimicrobial peptides (AMPs) are small proteins produced by some epithelial and immune cells and represent a crucial component of the innate immune system (Frew and Stock, 2011). They exhibit antibacterial, antifungal, antiviral and antiprotozoal activities and also possess anti-inflammatory and immunomodulatory activities, affect cell differentiation, angiogenesis, and wound healing (Mansour et al., 2014; Tribe, 2015; Yarbrough et al., 2015). AMPs play an important role in ensuring a healthy pregnancy and preventing complications (Frew and Stock, 2011). Namely, infections with bacteria, fungi, or viruses during pregnancy are associated with various adverse outcomes, such as miscarriage, eclampsia, premature rupture of membranes, premature delivery, growth restriction, and neonatal morbidity (Frew and Stock, 2011; Mei et al., 2019).

Unsurprisingly, the expression of several AMPs is induced in the case of intra-amniotic infection or inflammation (Figure 1). Among the most prominent AMPs are human β-defensins (HBD)1-3, which are expressed by multiple cells of hACM and are also found in the hAF (King et al., 2007a,b; Soto et al., 2007; Szukiewicz et al., 2016; Varrey et al., 2018; Para et al., 2020). Human neutrophil peptides (HNP)1-3, which also belong to the α defensin group, are present in the hAF of uncomplicated pregnancies but are more abundant during inflammation (Heine et al., 1998; Espinoza et al., 2003; Yoshio et al., 2003; Akinbi et al., 2004; Buhimschi et al., 2005). Moreover, antiproteases of the WAP (whey acidic protein) four-disulfide core (WFDC) family, elafin and secretory leukocyte peptidase inhibitor (SLPI), were found in hAEC and hCM, and SLPI was present in the hAF as well (Denison et al., 1999; Zhang et al., 2001; Akinbi et al., 2004; King et al., 2007a,b; Klaffenbach et al., 2011). Additionally, in the hAF, cathelicidin family members are responsive to the intra-amniotic infection or inflammation (Yoshio et al., 2003, 2005; Tambor et al., 2013). AMPs contribute significantly to the antimicrobial effects of fetal membranes and various approaches have been used to characterize and determine the efficiency of antimicrobial activity of fetal membranes and their derivatives against different pathogens.

## Different Approaches for Studying Antimicrobial Properties of Fetal Membranes

The use of fetal membranes with the aim of utilizing their medicinal properties was first described by Davis (1910), who used hAM as a skin graft. Although fetal membranes have been used in tissue engineering and regenerative medicine for almost a century, attention was brought to their antimicrobial properties for the first time in 1973 by Robson and Krizek (Robson et al., 1973; Lim, 2017). Over the next decades, researchers evaluated the antimicrobial properties of fetal membranes, using various approaches for the preparation of hACM, hAM and hCM and their derivatives and also several antimicrobial susceptibility testing methods. The studies analyzing antimicrobial properties of fetal membranes and different experimental approaches are summed up in the following subsections and in Tables 1, 2.

**TABLE 1:** Protocols for preparation of hACM, hAM, and hCM and their derivatives, which were used for investigation of antimicrobial activity. There are many variations in protocols for the preparation of fetal membranes to be used as an antimicrobial agent, which underlines the need for protocol standardization before considering clinical use.

Perinatal derivative	Preparation protocol	Use of antibiotics or other antimicrobial agents during preparation	References
hACM patches	hACM was separated from the placenta and rinsed in sterile saline. Patches of hACM were used immediately after separation or rinsed in 0.025% sodium hypochlorite, washed in saline, and stored at 4°C until use.	Yes	Robson and Krizek, 1973
hACM and hAM patches	hACM and hAM were separated from the placenta, rinsed in sterile saline, and stored in sterile isotonic saline solution at 4°C for up to 12 h before use.	No	Talmi et al., 1991
hACM patches	The hACM was obtained from the BioXclude, Snoasis Medical, Golden, CO, United States.	N/A	Ashraf et al., 2019
hACM patches	The hACM was obtained from the BioXclude, Snoasis Medical, Golden, CO, United States.	N/A	Palanker et al., 2019
hAM and hCM patches	hAM and hCM were separated from the placenta, rinsed in sterile saline, and used within 45 min after the separation.	No	Kjaergaard et al., 2001
hAM patches	hAM was separated from the placenta and rinsed in sterile PBS. The fresh hAM was used within the 4 h after the separation. The cryopreserved hAM was stored in sterile PBS containing 10% dimethylsulphoxide, 10% DMEM/F12, and 10% FBS at −80°C for 6 months. The freeze-dried hAM was pre-frozen for 30 min and lyophilized in a freeze-dryer at −55°C for 24 h and before use it was rehydrated in PBS for 2 h.	No	Tehrani et al., 2013
hAM and hCM patches	hAM and hCM were separated from the placenta, rinsed in sterile PBS, and cut into smaller pieces, which were incubated in sterile PBS for a maximum of 2 h at 4°C before use.	No	Zare Bidaki et al., 2012
hAM patches and hAM homogenate	hAM was separated from the placenta, rinsed in sterile PBS, and cut into smaller pieces. The patches of fresh hAM (f-hAM) were used within the 4 h after the separation. The patches of cryopreserved hAM (c-hAM) were stored in sterile PBS at −80°C for up to 10 weeks. The antibiotic-impregnated patches of c-hAM were rinsed with sterile PBS containing 50 μg/ml penicillin, 50 μg/ml streptomycin, 100 μg/ml neomycin, and 2.5 μg/ml amphotericin B and then stored in a culture medium supplemented with gentamicin (25 μg/ml). To prepare f-hAM and c-hAM homogenates, hAM was rinsed with sterile PBS, cut into pieces and then sterile PBS was added to patches of hAM (volume ratio 3 parts of PBS and 1 part of hAM) and homogenized in a homogenizer (Russell Hobbs, 21350-56, 300 W) for 3–4 min. Homogenate was stored at 4°C for a maximum of 6 h before use (f-hAM homogenate) or cryopreserved at –80 or at –20°C (c-hAM homogenate) for up to 10 weeks.	Yes for antibiotic-impregnated hAM patches	Ramuta et al., 2020
hAM patches and hAM homogenate	hAM was separated from the placenta, rinsed in sterile PBS, and cut into smaller pieces. The patches of f-hAM were used within the 4 h after the separation. The patches of c-hAM were stored in sterile PBS at −80°C for up to 1 month. To prepare f-hAM and c-hAM homogenates, hAM was rinsed with sterile PBS, cut into pieces and then sterile PBS was added to patches of hAM (volume ratio 3 parts of PBS and 1 part of hAM) and homogenized in a homogenizer (Russell Hobbs, 21350-56, 300 W) for 3–4 min. Homogenate was stored at 4°C for a maximum of 6 h before use (f-hAM homogenate) or cryopreserved at –80 or at –20°C (c-hAM homogenate) for up to 1 month. For testing the antibacterial activity of hAM homogenate on biomimetic urothelial *in vitro* models, the hAM homogenates were prepared in the culture media rather than PBS.	No	Ramuta et al., 2021
hAM homogenate	hAM was separated from the placenta, rinsed in sterile PBS, and cut into smaller pieces. Sterile PBS was added to pieces of hAM (volume ratio 3 parts of PBS and 1 part of hAM) and homogenized in a homogenizer (Russell Hobbs, 21350-56, 300 W) for 3–4 min. Homogenate was cryopreserved at –80°C for up to one year.	No	Šket et al., 2019
hCVAM patches	Human term placental tissues were obtained from commercial tissue agencies and processed by Osiris Therapeutics, Inc. (Columbia, MD, United States) following the proprietary manufacturing procedure. Namely, the hAM separated from the placenta within 36 h of the collection and incubated in the DMEM culture medium containing gentamicin, vancomycin, and amphotericin B for 18−48 h at 37°C and 5% CO_2_. Residual antibiotics were removed by washing the hAM with Dulbecco’s PBS and hAM was cut into pieces. Cryopreservation was performed by freezing hAM in DMSO containing cryoprotectant at a controlled cooling rate, according to the proprietary process developed by Osiris Therapeutics, Inc. Before use, hCVAM was thawed at a room temperature and rinsed in sterile PBS.	Yes	Mao et al., 2016
hCVAM conditioned medium	Human term placental tissues were obtained from commercial tissue agencies and processed by Osiris Therapeutics, Inc. (Columbia, MD, United States) following the proprietary manufacturing procedure. Namely, the hAM separated from the placenta within 36 h of the collection and incubated in the DMEM culture medium containing gentamicin, vancomycin, and amphotericin B for 18−48 h at 37°C and 5% CO_2_. Residual antibiotics were removed by washing the hAM with Dulbecco’s PBS and hAM was cut into pieces. Cryopreservation was performed by freezing hAM in DMSO containing cryoprotectant at a controlled cooling rate, according to the proprietary process developed by Osiris Therapeutics, Inc. Before use, hCVAM was thawed at room temperature and rinsed in sterile PBS. To prepare conditioned medium from hCVAM, the hCVAM was incubated for 6/22/24 h in DMEM, supplemented with 10% FBS (1 ml of culture medium per 4 cm^2^ of hCVAM) on a shaker.	Yes	Mao et al., 2017
hCVAM conditioned medium	Human term placental tissues were obtained from commercial tissue agencies and processed and Osiris Therapeutics, Inc. (Columbia, MD, United States) following the proprietary manufacturing procedure. Namely, the hAM separated from the placenta within 36 h of the collection and incubated in the DMEM culture medium containing gentamicin, vancomycin, and amphotericin B for 18-48 h at 37°C and 5% CO_2_. Residual antibiotics were removed by washing the hAM with Dulbecco’s PBS and hAM was cut into pieces. Cryopreservation was performed by freezing hAM in DMSO containing cryoprotectant at a controlled cooling rate, according to the proprietary process developed by Osiris Therapeutics, Inc. Before use, hCVAM was thawed at room temperature and rinsed in PBS. To prepare conditioned medium from hCVAM, the hCVAM was incubated for 4/20/24 h in DMEM, supplemented with 10% FBS (1 ml of culture medium per 4 cm^2^ of hCVAM) on a shaker.	Yes	Mao et al., 2018
hAM patches and hAM supernatant extract	hAM was prepared according to the protocol described by Talmi et al. (1991). To prepare acellular hAM, the hAM was digested for 15 min at 37°C by adding a mixture of 0.25% of trypsin and 0.06% EDTA, and then the cells were removed with a cell scraper. To prepare the hAM homogenate supernatant, the fresh intact hAM was rinsed in 0.01 mol/L PBS at 4°C for three times. Next, the hAM was cut into fragments and homogenized after adding 1:1 of 0.01 mol/L PBS. Then the mixture was sonicated, and PBS was added by 1:2 ratio and the solution was centrifuged at 2000 rpm, 4°C for 10 min. The supernatant was stored at 4°C and −20°C until use.	No	Wang et al., 2012
hAM and hCM extracts	hAM and hCM were separated from the placenta, rinsed in sterile PBS, cut into small pieces, and frozen using liquid nitrogen. Next, hAM was ground into fine particles using a mortar and pestle, mixed with PBS (1:1 ratio, wt/vol), and homogenized on ice for 1 h. The lysates were then centrifuged twice at 12,000 rpm at 4°C for 10 min and then the supernatants were filtered (0.22 μm pore size) to obtain the hAM extracts.	No	Yadav et al., 2017
hAM extract	hAM was separated from the placenta and rinsed in sterile PBS. To obtain the hAM extract, hAM was cut into pieces (1 × 1 cm), added to an equivalent volume of PBS and the hAM extract was obtained by sonicating the hAM on ice for 10 min with 80 W and 0.5 s cycle (Hielscher, Ultrasound Technology, Germany). The mixture was then centrifuged (800 rpm, 4 min) and the supernatant was stored.	No	Tehrani et al., 2017

*N/A, information not available.*

**TABLE 2:** The antimicrobial effect of fetal membranes and their derivatives on various microorganisms.

Microorganisms tested	Source of microorganism	Perinatal derivative	Antimicrobial effect (Yes/No)	References
**Bacteria**				
*Acinetobacter calcoaceticus*	Clinical strain	hCM patches	**Yes** in 87.5% of assays performed (in seven out of eight plates)	Kjaergaard et al., 2001
		hAM patches	**Yes** in 87.5% of assays performed (in seven out of eight plates)	
*Acinetobacter baumannii*	ATCC 33604	hAM patches	**No**	Ramuta et al., 2021
		hAM homogenate	**Yes** in 75% of all tested plates	
	ATCC 49466	hCVAM patches	**Yes**	Mao et al., 2016
	Clinical strain	hAM patches	**No**	Ramuta et al., 2021
		hAM homogenate	**Yes** in 75% of all tested plates	
*Aggregatibacter actinomycetemcomitans*	ATCC 33384	hACM patches	**Yes**	Ashraf et al., 2019
*Bacillus cereus*	ATCC 111778	hAM patches	**Yes** in 4.7% of all tested plates	Zare Bidaki et al., 2012
		hCM patches	**Yes** in 4.7% of all tested plates	
*Enterobacter sp.*	Environmental strain	hAM patches	**No**	Ramuta et al., 2020
		hAM homogenate	**Yes** in 100% of all tested plates	
*Enterobacter aerogenes*	ATCC 49469	hCVAM patches	**Yes**	Mao et al., 2016
*Enterobacter cloacae*	Clinical strain	hAM supernatant extract	**No**	Wang et al., 2012
*Enterococcus faecalis*	ATCC 51299	hAM patches	**No**	Ramuta et al., 2021
		hAM homogenate	**No**	
	Clinical strain	hCM patches	**Yes** in 87.5% of assays performed (in seven out of eight plates)	Kjaergaard et al., 2001
		hAM patches	**Yes** in 87.5% of assays performed (in seven out of eight plates)	
	Clinical strain	hAM patches	**No**	Ramuta et al., 2021
		hAM homogenate	**No**	
	Clinical strain	hAM supernatant extract	**Yes**	Wang et al., 2012
*Enterococcus faecium*	ATCC 51559	hCVAM patches	**Yes**	Mao et al., 2016
*Escherichia coli*	ATCC 25922	hAM patches	**No**	Tehrani et al., 2013
		hAM patches	**Yes** in 7% of all tested plates	Zare Bidaki et al., 2012
		hCM patches	**Yes** in 9.3% of all tested plates	
		hAM supernatant extract	**Yes**	Wang et al., 2012
		hAM extract	**Yes**	Tehrani et al., 2017
	DH5α, Invitrogen	hAM patches	**No**	Ramuta et al., 2020
		hAM homogenate	**Yes** in 100% of all tested plates	
	5 uropathogenic clinical strains	hAM patches	**No**	Ramuta et al., 2020
		hAM homogenate	**Yes** in 100% of all tested plates	
	Uropathogenic clinical strain	hAM patches	**Yes**	Šket et al., 2019
		hAM homogenate	**Yes**	
	Extended-spectrum β-lactamase positive; clinical strain	hAM patches	**No**	Ramuta et al., 2021
		hAM homogenate	**Yes** in 100% of all tested plates	
	Clinical strain (T3)	hAM patches	**Yes** in 90% of all assays (19 out of 21 plates) when using fresh hAM patches; in 58% of all assay (7 out of 12 plates) when using cryopreserved hAM patches and in 60% of all assays when using the freeze-dried hAM patches (9 out of 15 plates)	Tehrani et al., 2013
		hAM extract	**Yes**	Tehrani et al., 2017
	Clinical strain (T4)	hAM patches	**Yes** in 86% of all assays (12 out of 14 plates) when using fresh hAM patches; in 55% of all assay (6 out of 11 plates) when using cryopreserved hAM patches and in 67% of all assays when using the freeze-dried hAM patches (8 out of 12 plates)	Tehrani et al., 2013
		hAM extract	**Yes**	Tehrani et al., 2017
	Clinical strain	hAM supernatant extract	**No**	Wang et al., 2012
	Clinical strain	hCM patches	**Yes** in 87.5% of assays performed (in seven out of eight plates)	Kjaergaard et al., 2001
		hAM patches	**Yes** in 87.5% of assays performed (in seven out of eight plates)	
	N/A	hACM patches	**Yes**	Talmi et al., 1991
		hAM patches	**Yes**	
	N/A	hACM homogenate	**Yes**	Robson and Krizek, 1973
*Klebsiella pneumoniae*	ATCC 700603	hCVAM patches	**Yes**	Mao et al., 2016
	ATCC 700603	hAM patches	**Yes** in 4.7% of all tested plates	Zare Bidaki et al., 2012
		hCM patches	**Yes** in 9.3% of all tested plates	
	ATCC 700603	hAM patches	**No**	Ramuta et al., 2021
		hAM homogenate	**Yes** in 25% of all tested plates	
	Extended spectrum β lactamase-positive clinical strain	hAM patches	**No**	Ramuta et al., 2021
		hAM homogenate	**Yes** in 25% of all tested plates	
	Environmental strain	hAM patches	**No**	Ramuta et al., 2020
		hAM homogenate	**Yes** in 100% of all tested plates	
	N/A	hACM patches	**Yes**	Talmi et al., 1991
		hAM patches	**Yes**	
*Lactobacillus sp.*	Clinical strain	hCM patches	**Yes** in 87.5% of assays performed (in seven out of eight plates)	Kjaergaard et al., 2001
		hAM patches	**Yes** in 87.5% of assays performed (in seven out of eight plates)	
*Lactobacillus plantarum*	PTCC 1745	hAM patches	**Yes** in 4.7% of all tested plates	Zare Bidaki et al., 2012
		hCM patches	**No**	
*Morganella morganii*	Environmental strain	hAM patches	**No**	Ramuta et al., 2020
		hAM homogenate	**Yes** in 100% of all tested plates	
*Proteus mirabilis*	Environmental strain	hAM patches	**No**	Ramuta et al., 2020
		hAM homogenate	**Yes** in 100% of all tested plates	
	Clinical strain	hAM supernatant extract	**Yes**	Wang et al., 2012
	N/A	hACM patches	**Yes**	Talmi et al., 1991
		hAM patches	**Yes**	
*Providencia rettgeri*	Environmental strain	hAM patches	**No**	Ramuta et al., 2020
		hAM homogenate	**Yes** in 100% of all tested plates	
*Pseudomonas aeruginosa*	ATCC 15692	hCVAM patches	**Yes**	Mao et al., 2016
		hCVAM conditioned medium	**Yes**	Mao et al., 2018
		hCVAM conditioned medium	**Yes**	Mao et al., 2018
	ATCC 27853	hAM supernatant extract	**Yes**	Wang et al., 2012
		hAM extract	**Yes**	Tehrani et al., 2017
		hAM patches	**Yes** in 86% of all assays (12 out of 14 plates) when using fresh hAM patches; in 64% of all assays (7 out of 11 plates) when using cryopreserved hAM patches and in 58% of all assays when using the freeze-dried hAM patches (7 out of 12 plates)	Tehrani et al., 2013
		hAM patches	**No**	Ramuta et al., 2021
		hAM homogenate	**No**	
	ATCC 27883	hAM patches	**Yes** in 11.6% of all tested plates	Zare Bidaki et al., 2012
		hCM patches	**Yes** in 4.7% of all tested plates	
	Carbapenem-resistant clinical strain	hAM patches	**No**	Ramuta et al., 2021
		hAM homogenate	**No**	
	Clinical strain	hCM patches	**Yes** in 87.5% of assays performed (in seven out of eight plates)	Kjaergaard et al., 2001
		hAM patches	**Yes** in 87.5% of assays performed (in seven out of eight plates)	
	N/A	hACM patches	**Yes**	Robson and Krizek, 1973
		hACM homogenate	**No**	
	N/A	hACM patches	**Yes**	Talmi et al., 1991
		hAM patches	**Yes**	
*Serratia marcescens*	Environmental strain	hAM patches	**No**	Ramuta et al., 2020
		hAM homogenate	**No**	
		hAM patches	**No**	Šket et al., 2019
		hAM homogenate	**No**	
Coagulase-positive *Staphylococcus* sp.	N/A	hACM patches	**Yes**	Talmi et al., 1991
		hAM patches	**Yes**	
*Staphylococcus aureus*	ATCC 25923	hCVAM patches	**Yes**	Mao et al., 2016
		hCVAM conditioned medium	**Yes**	Mao et al., 2018
		hAM supernatant extract	**Yes**	Wang et al., 2012
		hAM extract	**Yes**	Tehrani et al., 2017
		hAM patches	**No**	Tehrani et al., 2013
		hCVAM conditioned medium	**Yes**	Mao et al., 2018
	ATCC 29213	hAM patches	**Yes** in 48.8% of all tested plates	Zare Bidaki et al., 2012
		hCM patches	**Yes** in 90.7% of all tested plates	
	Clinical strain	hCM patches	**Yes** in 87.5% of assays performed (in seven out of eight plates)	Kjaergaard et al., 2001
		hAM patches	**Yes** in 87,5% of assays performed (in seven out of eight plates)	
	Environmental strain	hAM patches	**No**	Ramuta et al., 2020
		hAM homogenate	**Yes** in 100% of all tested plates	
		hAM patches	**Yes**	Šket et al., 2019
		hAM homogenate	**Yes**	
Methicillin-resistant *Staphylococcus aureus*	NCTC 12493	hAM patches	**No**	Ramuta et al., 2021
		hAM homogenate	**Yes** in 100% of all tested plates	
	ATCC BAA-1720	hCVAM conditioned medium	**Yes**	Mao et al., 2018
	Clinical strain	hAM patches	**No**	Ramuta et al., 2021
		hAM homogenate	**Yes** in 100% of all tested plates	
	Clinical strain	hAM supernatant extract	**No**	Wang et al., 2012
*Staphylococcus epidermidis*	Clinical strain	hAM supernatant extract	**Yes**	Wang et al., 2012
*Staphylococcus haemolyticus*	Clinical strain	hAM supernatant extract	**Yes**	Wang et al., 2012
*Staphylococcus saprophyticus*	Clinical strain	hCM patches	**Yes** in 87.5% of assays performed (in seven out of eight plates)	Kjaergaard et al., 2001
		hAM patches	**Yes** in 87.5% of assays performed (in seven out of eight plates)	
	Environmental strain	hAM patches	**No**	Ramuta et al., 2020
		hAM homogenate	**Yes** in 100% of all tested plates	
*Shigella flexneri*	ATCC 12022	hAM patches	**Yes** in 16.3% of all tested plates	Zare Bidaki et al., 2012
		hCM patches	**Yes** in 9.3% of all tested plates	
Group B *Streptococcus*	Five clinical strains	hCM patches	**Yes** in 77.5% of assays performed (in 31 out of 40 plates)	Kjaergaard et al., 2001
		hAM patches	**Yes** in 77.5% of assays performed (in 31 out of 40 plates)	
*Streptococcus mutans*	ATCC 25175	hACM patches	**Yes**	Ashraf et al., 2019
*Streptococcus oralis*	ATCC 9811	hACM patches	**Yes**	Ashraf et al., 2019
*Streptococcus pneumoniae*	NCTC 7466	hAM extract	**Yes**	Yadav et al., 2017
		hCM extract	**Yes**	
	Serotype 3; ATCC 6303	hAM extract	**Yes**	Yadav et al., 2017
		hCM extract	**Yes**	
	Serotype 19A, 19F; ATCC 49619	hAM extract	**Yes**	Yadav et al., 2017
		hCM extract	**Yes**	
	Serotype 11; clinical strain	hAM extract	**Yes**	Yadav et al., 2017
		hCM extract	**Yes**	
	Clinical strain	hAM supernatant extract	**Yes**	Wang et al., 2012
*Streptococcus pyogenes*	ATCC 19615	hAM patches	**Yes** in 4.7% of all tested plates	Zare Bidaki et al., 2012
		hCM patches	**Yes** in 41% of all tested plates	
**Fungi**				
*Blastomyces albicans*	Clinical strain	hAM supernatant extract	**Yes**	Wang et al., 2012
*Fusarium solani*	Clinical strain	hAM supernatant extract	**Yes**	Wang et al., 2012
*Aspergillus fumigatus*	Clinical strain	hAM supernatant extract	**Yes**	Wang et al., 2012

*N/A, information not available.*

Interestingly, to the best of our knowledge, no studies focused solely on the antimicrobial activity of cells isolated from fetal membranes or their conditioned medium. The effect of fetal membranes-derived cells and their derivatives (conditioned medium, extract, supernatant) was only indirectly investigated by comparing the antimicrobial effect of intact and deepithelized hAM (Wang et al., 2012) and analyzing the amount of AMPs present in the extract or conditioned medium derived from fetal membranes (Mao et al., 2017; Tehrani et al., 2017).

### The Antimicrobial Activity of hACM Patches

Robson and Krizek (1973) investigated the effect of the human amnio-chorionic membrane (hACM) on the bacterial population of infected burns. They used hACM immediately after separation or rinsed it in sodium hypochlorite and stored it in saline at 4°C until use. On day 0 they inoculated full-thickness burn areas of 50 Sprague-Dawley rats with the overnight culture of *Pseudomonas aeruginosa.* After 5 days, the 38 rats that survived the wound sepsis underwent surgical removal of the burn eschar. Afterward, the three areas of the burn were treated as follows: one had hACM sutured into the defect; another had a piece of human skin (approximately 0.35 mm in thickness) sutured into the defect; the third area was left untreated as a control. The positions chosen for each type of treatment were randomized on the various rats. The hACM and skin grafts were replaced by fresh material every 48 h. At the time of each change, tissue biopsies were obtained for quantitative and qualitative bacteriology. Both treatments resulted in a decrease of the bacterial count in virtually all animals, but the degree of decrease was significantly greater when hACMs were applied. They performed another experiment, in which the overnight cultures of *P. aeruginosa* and *Escherichia coli* were incubated with hACM homogenate or a homogenate of split-thickness human skin. Interestingly, after 18–24 h of incubation, no antibacterial effect of either homogenate was detected. In the last part of this study ten patients with deep partial-thickness or full-thickness burns were treated with topical antibacterial agents and then biological dressings, namely hACM or human skin grafts, were applied. Biological dressings were replaced every 48 h and were used in full-thickness burns until replaced with autografts and in partial-thickness burns until re-epithelization occurred. Application of hACM and human skin grafts resulted in a further decrease of the bacterial count (Robson and Krizek, 1973). Unfortunately, it is not clear which hACM preparation (fresh hACM or hACM that was rinsed in sodium hypochlorite and stored at 4°C) was used for each experiment and this is a large shortcoming of this study since we cannot conclude whether the antimicrobial effect stems from the intrinsic antimicrobial properties of hACM or the remnants of sodium hypochlorite that remained in the hACM after rinsing. However, this study served as a meaningful booster for further research.

Talmi et al. (1991) performed a modified disk-diffusion susceptibility test, in which they evaluated the antimicrobial effect of fresh hACM, hAM, and synthetic polyurethane-based membranes on coagulase-positive *Staphylococcus* sp., *E. coli*, *Klebsiella pneumoniae*, *P. aeruginosa*, and *Proteus mirabilis*. They demonstrated that all membranes inhibited bacterial growth directly under the membranes but there was no inhibition zone, which led researchers to the conclusion that the antimicrobial effect is the result of the adherence of the membranes to the surface (Talmi et al., 1991).

Ashraf et al. (2019) used a commercially available hACM (BioXclude, Snoasis Medical, Golden, CO, United States) and investigated its antimicrobial activity. They inoculated disks of hACM with *Aggregatibacter actinomycetemcomitans*, *Streptococcus mutans*, and *Streptococcus oralis*, collected the samples after 12 and 24 h, and quantified bacterial growth. Their results show that hACM inhibited the growth of all tested bacterial strains at both time points and hACM was as effective as tetracycline (62 μg/ml) that was used as a control (Ashraf et al., 2019). Similarly, Palanker et al. (2019) also used the commercially available hACM (BioXclude), which they inoculated with *Streptococcus gordonii*. Their results showed that the hACM significantly inhibited bacterial growth at 8, 24, and 48 h of incubation. As a negative control, they used porcine pericardium collagen membrane, which did not inhibit bacterial growth (Palanker et al., 2019).

### The Antimicrobial Activity of the hAM and hCM Patches

Kjaergaard et al. (2001) investigated the antibacterial activity of fresh hCM and hAM on clinical isolates of hemolytic *Streptococcus* group A, *Staphylococcus aureus*, *Staphylococcus saprophyticus*, *Enterococcus faecalis*, *E. coli*, *P. aeruginosa*, *Acinetobacter calcoaceticus*, and *Lactobacillus* sp. They placed fresh hCM and hAM patches, washed only with saline, on bacteria-inoculated agar plates, or suspended them in agar. After 24 h of incubation, no colonies were found underneath the hCM or hAM patches and there was a narrow inhibition zone (1 mm) around the edge of the membranes in 77.5% (in 31 out of 40 plates per tested strain) of the group B *Streptococcus*-inoculated agar plates and in 87.5% (in seven out of eight plates per tested strain) of agar plates inoculated with the rest of aforementioned bacteria. Importantly, after 24 h of incubation, the researchers removed the hCM and hAM and incubated the plates for additional 24 h and no reversal of inhibition was observed. Likewise, the fetal membranes immersed in agar also inhibited bacterial growth beneath the membranes. On the other hand, when hCM and hAM were placed in inoculated broth medium, the researchers stated that only hCM showed a marginal inhibition of bacterial growth, while no inhibition was observed with hAM (Kjaergaard et al., 2001).

Wang et al. (2012) investigated the antimicrobial activity of hAM patches on the (1) reference strains of *S. aureus*, *P. aeruginosa*, and *E. coli*, (2) clinical strains of *Streptococcus pneumoniae*, *Staphylococcus epidermidis*, *Staphylococcus haemolyticus*, and *P. mirabilis*, (3) multidrug-resistant clinical strains of *E. faecalis*, *Enterobacter cloacae*, *E. coli*, and methicillin-resistant *S. aureus* (MRSA) and (4) fungal strains of *Blastomyces albicans*, *Fusarium solani*, and *Aspergillus fumigatus*. They examined the antimicrobial activity of fresh hAM and fresh deepithelized hAM patches and demonstrated that the application of fresh hAM resulted in a significant bacteriostatic ring around the membrane, but the application of the fresh deepithelized hAM patch had not resulted in a bacteriostatic ring, which indicated that antimicrobial molecules are produced by the hAEC (Wang et al., 2012).

Tehrani et al. (2013) compared the antimicrobial efficiency of fresh hAM, cryopreserved hAM, and freeze-dried hAM patches against reference strains of *S. aureus*, *P. aeruginosa*, *E. coli*, and two clinical strains of *E. coli* using a modified disk diffusion method. When fresh, cryopreserved, and freeze-dried hAM patches were applied to agar plates inoculated with the reference strain of *P. aeruginosa* and both clinical strains of *E. coli*, the bacterial growth was inhibited under all hAM and there was also a narrow inhibition zone (maximum of 5 mm) around the hAM. Moreover, the range of the antimicrobial effect varied depending on the preservation method and bacterial strain. On average, the fresh hAM induced antimicrobial effect in 88% of all plates (43 out of 49 plates) in all susceptible bacterial strains, while the cryopreserved hAM and freeze-dried hAM induced antimicrobial effect in 59% (20 out of 34 plates) and 62% of all plates (24 out of 39 plates), respectively. Furthermore, the fresh hAM induced the largest inhibition zones. On the other hand, none of the hAM preparations induced the antimicrobial effect in the reference strains of *S. aureus* and *E. coli*. The authors also measured the amount of elafin, in the extracts of fresh, cryopreserved, and freeze-dried hAM patches. Their results showed that cryopreservation and freeze-drying significantly decreased the amount of elafin while the antimicrobial effect was still present, which indicates that other antimicrobial molecules and also extracellular matrix may contribute to the antimicrobial effect of hAM (Tehrani et al., 2013). In another study, Tehrani et al. (2017) demonstrated that the orientation of hAM (epithelial or mesenchymal side up) in the modified disc diffusion method does not affect the antimicrobial efficiency of hAM patches. Moreover, they exposed hAM patches to cytokine interleukin 1β (IL-1β) and demonstrated that IL-1β induces a higher level of AMP secretion by the hAM cells, namely elafin, HBD-2, HBD-3, and cathelicidin LL-37 (Tehrani et al., 2017).

Zare Bidaki et al. (2012) placed fresh patches of hAM and hCM on agar plates inoculated with the following bacterial strains: *E. coli*, *Bacillus cereus*, *K. pneumoniae*, *Streptococcus pyogenes*, *S. aureus*, *Shigella flexneri*, and *Lactobacillus plantarum*. Their results showed that the application of hAM patches resulted in an inhibition zone in 5% (*K. pneumoniae*, *S. pyogenes*, *L. plantarum*) to 49% (*S. aureus*) of tested plates, while the application of hCM patches resulted in an inhibition zone in 5% (*S. pyogenes, B. cereus*) to 91% (*S. aureus*) of tested plates, but did not cause an inhibition zone in *L. plantarum* (Zare Bidaki et al., 2012).

The antimicrobial activity of hAM patches was also investigated by our research group. We prepared fresh and cryopreserved patches of hAM and tested their antimicrobial activity against 14 strains of most common uropathogenic bacteria (5 clinical strains of uropathogenic *E. coli* (UPEC) and 1 reference or environmental strain of *S. aureus*, *E. coli*, *S. saprophyticus*, *Morganella morganii*, *Providencia rettgeri*, *K. pneumoniae*, *P. mirabilis*, *Serratia marcescens*, *Enterobacter* sp.) (Ramuta et al., 2020) and 11 strains of multidrug-resistant bacteria associated with urinary tract infections (reference and clinical strains of MRSA, extended-spectrum β-lactamases (ESBL) producing *E. coli* and *K. pneumoniae*, vancomycin-resistant Enterococci, carbapenem-resistant *Acinetobacter baumannii*, and *P. aeruginosa*) (Ramuta et al., 2021). The fresh and cryopreserved hAM patches were embedded into Muller-Hinton soft agar inoculated with the aforementioned strains and after 24 h of incubation, the antimicrobial effect was evaluated. Interestingly, the application of neither fresh nor cryopreserved patches of hAM resulted in the antimicrobial effect in any of the tested strains. Therefore, we further evaluated the antimicrobial activity of hAM that was prepared and stored according to the standard procedure for use of hAM in ophthalmology (Jirsova and Jones, 2017). Specifically, hAM was during preparation briefly washed with antibiotics and antimycotics (50 μg/ml penicillin, 50 μg/ml streptomycin, 100 μg/ml neomycin, 2.5 μg/ml amphotericin B) and then stored in a culture medium supplemented with gentamicin (25 μg/ml). So prepared membrane was applied to two clinical strains of UPEC, one strain of *S. marcescens*, and two gentamicin-resistant UPEC strains. The antimicrobial effect was observed with all tested wild-type strains, but no antimicrobial effect was observed in the case of gentamicin-resistant UPEC strains. We demonstrated that the observed antimicrobial effect was due to the presence of gentamicin in the hAM patches. Further, experiments with gentamicin-sensitive UPEC strains revealed that even extensive rinsing of hAM patches in PBS only decreased the concentration of the antibiotics in the hAM patches, but did not completely remove them. Therefore, we suggested the high retention of antibiotics in hAM patches could be attributed to its ultrastructure, as the unique structure and composition of the hAM stroma might enable entrapment of antibiotics inside the extracellular matrix. Hence, hAM patches have great potential to be used as drug delivery tools (Ramuta et al., 2020).

Mao et al. (2016) used patches of commercially available so-called human cryopreserved viable amniotic membrane (hCVAM; Osiris Therapeutics Inc., Columbia, MD, United States; Table 1) and placed them on the agar plates, which were then inoculated with *Enterococcus faecium*, *S. aureus*, *K. pneumoniae*, *A. baumannii*, *P. aeruginosa*, and *Enterobacter aerogenes*. After 18 h bacteria were eluted from samples and quantified. Next, they incubated patches of hCVAM in 1 ml of culture medium containing bacteria for 24 h and then quantified the number of bacteria. Using both methods they demonstrated that hCVAM patches had an antibacterial effect on all tested strains (Mao et al., 2016).

In the next study, Mao et al. (2017) prepared the conditioned medium by incubating the hCVAM patches in culture medium for 6, 22, and 24 h. They showed that the conditioned medium had an antimicrobial effect on *P. aeruginosa*, *S. aureus*, and MRSA. In addition, they also showed that the antimicrobial activity was mediated via the secretion of soluble factors by viable cells in hCVAM, as the antimicrobial effect in the conditioned medium prepared from the air-dried devitalized membrane was significantly lower. After analyzing the hCVAM conditioned medium obtained after 6 and 22 h of incubation, they showed that the accumulation of antimicrobial factors in the conditioned medium is time-dependent as the conditioned medium obtained after 22 h of incubation had the largest antimicrobial effect and the largest expression of HBD-2 and HBD-3, histone H2B and SLPI. Moreover, by employing immunoprecipitation they removed HBD-2 and HBD-3 from the conditioned medium and demonstrated that HBD-2 and HBD-3-depleted hCVAM conditioned medium has reduced antibacterial activity (Mao et al., 2017).

The same research group investigated also the effect of hCVAM-derived conditioned medium on the *S. aureus* and *P. aeruginosa* biofilm formation. They demonstrated that the formation of biofilm was reduced in the presence of the hCVAM-derived conditioned medium. Moreover, they confirmed this result with the *ex vivo* experiment. Namely, they incubated sterile porcine dermal tissue pieces in hCVAM-conditioned medium or in assay medium (control) for 4 h at room temperature with gentle shaking. Then they inoculated the surface of porcine dermal tissue saturated with hCVAM- conditioned medium or the assay medium with *P. aeruginosa* and performed a quantitative assessment of the biofilm after 48 h of incubation. They showed that the biofilm formation on porcine dermal tissues saturated with the hCVAM-conditioned medium was reduced by 97% in comparison to the biofilm formation on the dermal tissues saturated with assay medium. Interestingly, no significant inhibition of *S. aureus* or MRSA biofilm formation was observed in dermal tissues treated with hCVAM-derived conditioned medium (Mao et al., 2018).

The studies by Mao et al. (2016, 2017, 2018) importantly contribute to basic knowledge regarding the antimicrobial activity of hCVAM patches and conditioned medium against several clinically important pathogens. However, it is important to note that the procedure for preparation of hCVAM includes incubation in a culture medium supplemented with antibiotics and antimycotics (Duan-Arnold et al., 2015) and since the authors did not investigate whether there were any remnants of the antibiotics and antimycotics in the hCVAM after rinsing, it is impossible to know whether the antimicrobial activity of hCVAM can be solely attributed to the hAM-derived AMPs.

### The Antimicrobial Activity of the hAM Homogenate

Homogenate is a mixture of extracellular matrix and cells that has been obtained by mechanical disruption. Our research group prepared fresh (f-hAM) and cryopreserved hAM homogenate (c-hAM) and evaluated their antimicrobial activity against 14 strains of the most common uropathogenic bacteria (Ramuta et al., 2020) and 11 strains of multidrug-resistant bacteria associated with urinary tract infections (Ramuta et al., 2021). Using the antimicrobial efficiency assay with f-hAM and c-hAM homogenates on agar plates, we showed that hAM homogenates caused an inhibition zone in all performed tests in 16 out of 25 tested strains (five clinical strains of UPEC, a clinical strain of β lactamase-producing *E. coli*, laboratory strain of *E. coli* DH5α, reference and clinical strain of methicillin-resistant *S. aureus*, environmental strains of *S. saprophyticus*, *S. aureus*, *M. morganii*, *P. rettgeri*, *K. pneumoniae*, *P. mirabilis*, *Enterobacter* spp.), while in two strains they caused an inhibition zone in 25% of performed tests (reference and clinical strains of β lactamase-producing *K. pneumoniae*), and in two strains they caused an inhibition zone in 75% of performed tests (reference and clinical strains of *A. baumannii*). Moreover, in five strains the f-hAM and c-hAM homogenates did not cause an inhibition zone in any of the performed tests (reference and clinical strains of *P. aeruginosa* and *E. faecalis* and environmental strain of *S. marcescens*). Next, we demonstrated that the manner of preparation and storage of hAM homogenate affects the range of its antimicrobial activity. Namely, the application of f-hAM homogenates resulted in the largest diameter of the inhibition zone, followed by (1) c-hAM homogenates that were stored for 10 weeks at –20°C, (2) c-hAM homogenates that were stored for 1 week at –80°C and (3) c-hAM homogenates that were stored for 10 weeks at –80°C. Application of larger volumes (10 μl) of hAM homogenates also resulted in a larger inhibition zone than an application of smaller volumes (5 μl) of hAM homogenates. In another study (Šket et al., 2019) we investigated the antimicrobial activity of c-hAM homogenate in bacteria-inoculated liquid culture medium and we demonstrated that c-hAM homogenate, when twofold diluted had a bacteriostatic effect on UPEC and *S. aureus* and when it was diluted fourfold or eightfold had a bactericidal effect *S. aureus* strain. Moreover, in accordance with our experiments on agar plates, the c-hAM homogenate had no antimicrobial effect on *S. marcescens* in a liquid culture medium.

To test whether the c-hAM homogenate causes such robust antimicrobial effect also in a more complex microenvironment, we evaluated its antimicrobial activity in the MRSA-infected biomimetic *in vitro* models of normal and cancerous urinary bladder urothelia. We demonstrated that even a short-term incubation (3 h) in hAM homogenate significantly decreased the number of bacteria, but it did not affect the viability, number, and ultrastructure of urothelial cells (Ramuta et al., 2021).

### The Antimicrobial Activity of the hAM and hCM Supernatants and/or Extracts

Wang et al. (2012) investigated the antimicrobial activity of human amniotic homogenate supernatant (HAHS). To prepare HAHS, fragments of hAM have been sonicated, centrifuged and then the supernatant was obtained. HAHS were tested on the (1) reference strains of *S. aureus*, *P. aeruginosa*, and *E. coli*, (2) clinical strains of *S. pneumoniae*, *S. epidermidis*, *S. haemolyticus* and *P. mirabilis*, (3) multidrug-resistant clinical strains of *E. faecalis*, *E. cloacae*, *E. coli* and MRSA and (4) fungal strains of *B. albicans*, *F. solani*, and *A. fumigatus*. They reported that HAHS induced antimicrobial effect in all three reference strains, *S. epidermidis*, *P. mirabilis*, *S. pneumoniae*, *E. faecalis*, *F. solani*, *B. albicans*, and *A. fumigatus*, but did not induce an antimicrobial effect in multidrug-resistant *E. cloacae*, *E. coli* and MRSA. Moreover, they also demonstrated the stable antimicrobial characteristics of HAHS, since changes in pH (pH values 5.0, 6.0, 7.0, 8.0, 9.0), temperature (−20, 4, 20, 60°C) and storage time (1, 3, 7, 14, 28, 56 days at 4 °C) of HAHS did not significantly alter the antimicrobial efficiency of HAHS (Wang et al., 2012).

Yadav et al. (2017) prepared hAM and hCM extracts by grounding the frozen fragments of hAM and hCM using a mortar and pestle, mixing the particles with PBS, centrifuging the mixture and obtaining the supernatant that was filter sterilized. The so-called hAM and hCM extracts were used for evaluation of their antibacterial and antibiofilm activities against *Streptococcus pneumoniae* on an *in vitro* biofilm model and *in vivo* otitis media rat model. They demonstrated that the hAM and hCM extracts inhibited the growth of *S. pneumoniae* in planktonic form and decreased their microbial biomass in biofilms. Moreover, they also showed that biofilms that grew in the hAM and hCM extract were thin, scattered, and unorganized, and importantly, the hAM and hCM extracts strongly reduced the microbial biomass in the pre-established pneumococci biofilms and had a bactericidal effect. They also combined the hAM and hCM extracts with penicillin and streptomycin and showed that the hAM and hCM extracts together with antibiotics had a synergistic effect against *S. pneumoniae*. In the *in vivo* model, the hAM extract significantly reduced bacterial colonization in the rat middle ear. The authors also performed proteomics analysis and showed that the hAM and hCM extracts contain ribonucleases (ribonuclease T2, ribonuclease K6, ribonuclease 7, ribonuclease H2, ribonuclease pancreatic, ribonuclease 5) and antimicrobial peptides lactoferricin, lysozyme, dermcidin, granulysin, the proteins S100-A9 and S100-A8, β-2 microglobulin, antileukoproteinase, histones H2B type 1-D, type 1-O, HRA-V and H1-4 and HBD-3 (Yadav et al., 2017).

The antimicrobial activity of hAM extract was investigated also by Tehrani et al. (2017). Their results showed that the application of hAM extract resulted in a decrease in the number of colonies of *P. aeruginosa*, *E. coli*, and *S. aureus* (Tehrani et al., 2017).

## Translation of Fetal Membranes From Preclinical to Clinical Use: What Remains to Be Done?

Studies have shown that fetal membranes and their derivatives have broad-spectrum antimicrobial activity against a plethora of Gram-positive and Gram-negative bacteria and even some fungi. While these results are very promising, especially as they demonstrate that even some of the multidrug-resistant bacteria are susceptible to the antimicrobial activity of fetal membranes, there are several considerations to be taken into account when planning future studies and eventual translation from bench to bedside.

### Standardization of Sample Preparation

An overview of studies investigating the antimicrobial activity of fetal membranes and their derivatives showed that in numerous cases various authors present conflicting results using the same bacterial species or even the same bacterial strains. That illustrates the need for standardization of hACM, hAM, and hCM preparation as we can conclude from these studies that even minor differences in protocols account for diverse outcomes (Tables 1, 2). Therefore, to enable comparison of results among various studies, it is of the utmost importance that future studies report on the protocols for preparation and storage meticulously, including the information regarding rinsing (which buffer was used, how many times the fetal membrane was washed), storage times and temperatures, centrifugation specifications, sonication specifications, buffer and culture medium composition, etc. Moreover, going forward, research on antimicrobial properties of fetal membranes and their derivatives must be performed using only those that did not come into contact with antibiotics during preparation and storage. This is very important due to hAM’s capability for drug retention (Kim et al., 2001; Mencucci et al., 2006; Resch et al., 2011; Yelchuri et al., 2017; Sara et al., 2019; Ramuta et al., 2020) and it is otherwise impossible to conclude how much of the antimicrobial effect can be attributed to fetal membranes’ innate AMPs. Additionally, to be able to compare the antimicrobial effect of various hACM, hAM, and hCM-derived preparations, it will also be necessary to employ the same antimicrobial susceptibility testing methods. It will also be necessary to establish clinical laboratory standards for susceptibility testing of infectious agents to fetal membrane-derived preparations, similar to the Clinical & Laboratory Standards for antibiotics (Humphries et al., 2018). That would allow infectious agents to be characterized as “susceptible,” “intermediate” or “resistant” to various hACM, hAM, and hCM-derived preparations.

### Is Antimicrobial Activity of hACM Superior to hAM or hCM Alone?

If we wish to progress from bench to bedside, future studies will have to ascertain which fetal membrane-derived preparations at which concentrations provide the best clinical outcome. The review of the current literature does not offer a definitive answer which fetal membrane or fetal membrane-derived preparation provides the best antimicrobial effect. Namely, the hACM, hAM, and hCM have all been shown to possess antimicrobial properties, but to the best of our knowledge, no study compared the antimicrobial effect of these preparations on the same bacterial strains in the same experimental setting, it is therefore impossible to conclude which preparation is superior. However, until now, most studies investigating the antimicrobial properties of fetal membranes have been performed using hAM and since it has many properties that are beneficial in regenerative medicine, such as immunomodulatory properties (Magatti et al., 2016, 2018; Wassmer and Berishvili, 2020), promotion of epithelization and decrease of scarring (Koizumi et al., 2000; Gicquel et al., 2009; SantAnna et al., 2016), the translation from pre-clinic to clinic might be most swift for hAM. However, further research projects, which would address antimicrobial activity of hACM and hCM in the same experimental settings as were done for hAM, are highly recommended.

### The Need for More *in vivo* Studies

The majority of what we know regarding the antimicrobial properties of hACM, hCM, and hAM comes from *in vitro* studies. While these are of immense importance as they significantly contribute to our understanding of the mechanism of antimicrobial activity of fetal membranes, and their derivatives, there is a great deficiency of the *in vivo* studies, especially on large animals, which would allow us to evaluate the antimicrobial properties in a more complex (micro)environment. Moreover, the *in vivo* studies will also provide much-needed information regarding the safety of hACM, hCM, hAM, and their derivatives.

### Donor Selection

Future research should also focus on donor selection. Currently, most fetal membranes used for research are obtained from healthy donors that tested negative for HIV, hepatitis B, and syphilis and underwent a Cesarean section at a gestation age of 38–40 weeks. To reach optimal results it would be necessary to evaluate how the age and the lifestyle of the donor, gestation age, and manner of delivery (vaginal vs. Cesarean) affect the AMP production and secretion.

### The Possibility of Antimicrobial Resistance

Another aspect to consider when evaluating the potential of fetal membranes and their derivatives to be used as antimicrobial agents is the possibility of microbes developing resistance to antimicrobial molecules derived from fetal membranes. Therefore, future studies should focus also on assessing the risk of development and spread of microbial resistance to fetal membrane-derived antimicrobials, since that would pose an immense threat to the health of pregnant women and their developing children worldwide.

## Conclusion

To conclude, the use of fetal membranes has many advantages, since they provide a valuable source of cells and extracellular matrix with regenerative properties, they are immune-privileged tissue and importantly, their use is ethically acceptable (Lim, 2017). On the other hand, the use of fetal membranes comes with some challenges, such as donor variability, lack of standardized protocols for preparation, and limited shelf life. However, in the last decades, the number of studies investigating the properties and potential clinical use of fetal membranes is increasing, which led to a broadening of basic knowledge in the field of fetal membranes. Moreover, future studies will provide additional insight that will enable us to take advantage of various beneficial properties of fetal membranes and overcome the current challenges.

## Author Contributions

TŽR and MEK: conceptualization. TŽR: visualization. TŽR and TŠ: writing – original draft preparation. TŽR, TŠ, MSE, and MEK: writing – review and editing. All authors: read and approved the final version of the manuscript.

## Conflict of Interest

The authors declare that the research was conducted in the absence of any commercial or financial relationships that could be construed as a potential conflict of interest.
